# Assessment of Platelet Response to Aspirin Therapy and Hemocompatibility-Related Adverse Events in HeartMate 3 Left Ventricular Assist Device Recipients

**DOI:** 10.3390/jcm13237234

**Published:** 2024-11-28

**Authors:** Hebe Al Asadi, Theodor Abart, Caroline Schwarz, Roxana Moayedifar, Anne-Kristin Schaefer, Christiane Marko, Barbara Messner, Daniel Zimpfer, Julia Riebandt, Thomas Schlöglhofer

**Affiliations:** 1 Department of Cardiac Surgery, Medical University of Vienna, 1090 Vienna, Austria; hebe.alasadi@meduniwien.ac.at (H.A.A.);; 2 Ludwig Boltzmann Institute for Cardiovascular Research, 1090 Vienna, Austria; 3 Center for Medical Physics and Biomedical Engineering, Medical University of Vienna, 1090 Vienna, Austria

**Keywords:** left ventricular assist device, mechanical circulatory support, hemocompatibility-related adverse events, antiplatelet therapy, platelet response, platelet sensitivity assays, point-of-care

## Abstract

**Background**: Patients with a HeartMate 3 (HM3) left ventricular assist device (LVAD) typically receive anticoagulation and antiplatelet therapy. The HM3 has shown a marked reduction in hemocompatibility-related adverse events (HRAEs) like stroke, bleeding, and pump thrombosis. This study evaluated whether aspirin (ASA) response influences HRAE incidence and if ASA sensitivity changes over time in HM3 recipients. **Methods**: This single-center, cross-sectional study included 32 HM3 patients (age: 59.0 ± 10.0 years, 15.6% female). ASA sensitivity was assessed twice using the VerifyNow assay, with ASA resistance defined by ASA reactivity units (ARU) > 550. The primary endpoint was HRAE incidence in ASA responders vs. non-responders over two consecutive follow-ups; the secondary endpoint was temporal changes in ASA resistance. **Results**: At the first follow-up, 13 (40.6%) patients were ASA-resistant, and 8 (28.6%) were resistant at the second follow-up, without significant change (*p* = 0.22). ASA non-responders and responders had similar ASA doses and baseline characteristics. No significant difference in HRAE incidence was found between ASA non-responders and responders (0.0% vs. 15.8%, *p* = 0.14), and no additional HRAEs occurred during follow-up. **Conclusions**: ASA resistance varied considerably among HM3 patients without significant temporal changes, and the demonstrated excellent hemocompatibility supports recent evidence that ASA may have a limited role in the antithrombotic regimen for HM3 recipients.

## 1. Introduction

Left ventricular assist device (LVAD) therapy has emerged as a widely accepted treatment for end-stage heart failure, serving as either a bridge to transplant or as destination therapy [1]. As previous studies have shown, mechanical circulatory support (MCS) has consistently enhanced patient survival and quality of life, largely attributed to advancements in device design [2]. The centrifugal-flow HeartMate 3 (HM3) (Abbott, Chicago, IL, USA) has demonstrated a significant decrease in the incidence of hemocompatibility-related adverse events [3] (HRAEs) such as strokes, bleeding, and pump thrombosis compared to its predecessor, the axial-flow HeartMate II (HM II), in the randomized controlled MOMENTUM 3 trial, as demonstrated in both the 2-year [2] and 5-year follow-up studies [4].

To prevent HRAEs with contemporary MCS [5], typically, anticoagulation for long-term LVAD patients consists of a vitamin K antagonist (VKA), such as warfarin or phenprocoumon [6,7], along with aspirin/acetylsalicylic acid (ASA), as antiplatelet therapy [2,6]. ASA inhibits platelet activation by irreversibly acetylating serine 530 of cyclooxygenase (COX)-1, a crucial enzyme in synthesizing thromboxane A2 from arachidonic acid. This inhibits platelet activation, preventing the formation of thromboxane A2 and subsequent platelet activation [8,9]. According to the 2023 International Society for Heart and Lung Transplantation (ISHLT) Guidelines for Mechanical Circulatory Support [10], a daily dose of 81–325 mg ASA therapy is recommended in addition to VKA (with an INR target range of 2.0–3.0) to reduce the thrombotic risk. Previously, Saeed et al. [11] found no differences in survival free from hemorrhagic or thrombotic events between HM3 usual-dose (325 mg) and low-dose (81 mg) ASA groups. A recent retrospective study by Yi et al. [12] analyzed 81 HM3 patients who received either 100 mg/day ASA in combination with warfarin or warfarin alone, finding that the ASA group had significantly higher odds of bleeding events (32.1% vs. 7.1%, *p* = 0.013), while the odds of thromboembolic events remained similar [12].

In addition, multiple studies have reported variations in the response to ASA therapy, also known as ASA resistance, indicating that 5–45% of patients may not experience the expected platelet-inhibiting effect [13,14,15]. 

Various methods and platelet sensitivity assays, including light transmission aggregometry (LTA) as the gold standard [16], VerifyNow, Platelet Function Analyzer (PFA-200), and thromboelastography (TEG), have been used to measure platelet inhibition and monitor the patient response to ASA therapy [17,18]. All these methods operate on similar mechanisms, by measuring the response of platelet-rich plasma (PRP) or whole blood to external aggregating agonists, such as adenosine-diphosphate (ADP), arachidonic acid (AA), collagen, and/or epinephrine (EPI) [18]. While LTA employs PRP as a medium and measures the increase in light transmission through a sample, which becomes clearer if more platelets clot together [18], the primary advantage of VerifyNow point-of-care (POC) is that it uses whole blood, eliminating the need for processing or specialist laboratory assistance [17]. VerifyNow ASA responsiveness is determined similarly to LTA by a turbidimetric-based optical-based assay test within a few minutes [18,19]. PFA-200 simulates high shear stress by propelling whole blood through a steel capillary, which contains a collagen-coated membrane, and an aperture infused with EPI or ADP. It measures the time it takes to occlude the aperture, known as the closure time, where lower closure times indicate higher platelet activation [17,18]. With TEG, a whole blood sample rotates in a cup around a fixed pin suspended by a torsion wire. Shear stress from the rotation and the infusion of reagents leads to clot formation, and these changes in viscosity cause motion in the pin, which is then detected [18].

Alongside the variations in ASA sensitivity among patients, temporal changes in ASA sensitivity have been demonstrated. DeNino et al. [20] presented two cases where patients exhibited an ASA response upon discharge from the hospital but were found to be aspirin-resistant during rehospitalization when retested. In another recent study involving 28 LVAD patients, only 7% were aspirin-resistant at baseline, but the number of ASA non-responders increased to 32% three months post-implantation [21]. Given the near elimination of thrombosis [2,4] with the HM3 but the persistent occurrence of bleeding events [8], notably gastrointestinal bleeding (GIB), in LVAD patients, the question arises whether differences and fluctuations in ASA sensitivity are associated with HRAEs. Therefore, the primary objective of this study was to investigate whether the response to ASA therapy, as determined by VerifyNow POC testing, impacts the incidence of HRAEs during HM3 support. Secondary endpoints included survival stratified by ASA response, as well as temporal changes in ASA sensitivity and potential correlations with demographics, comorbidities, and other medications.

## 2. Materials and Methods

### 2.1. Study Population

This cross-sectional, observational, single-center cohort study included 32 patients with HM3 LVAD, implanted between 2019 and 2021, all aged at least 18 years. The study protocol was approved by the Institutional Review Board (identification number: EK/2460/2020, ClinicalTrials.gov Identifier: NCT06152562), and all participants provided written informed consent. The institutional anticoagulation and antiplatelet therapy guidelines [6,7] comprised VKA (phenprocoumon) therapy after the removal of chest tubes (INR target range 2.0–2.5), and ASA therapy was initiated on the 3rd postoperative day (POD) at a dose of 100 mg/day unless contraindicated.

**Figure 1:**
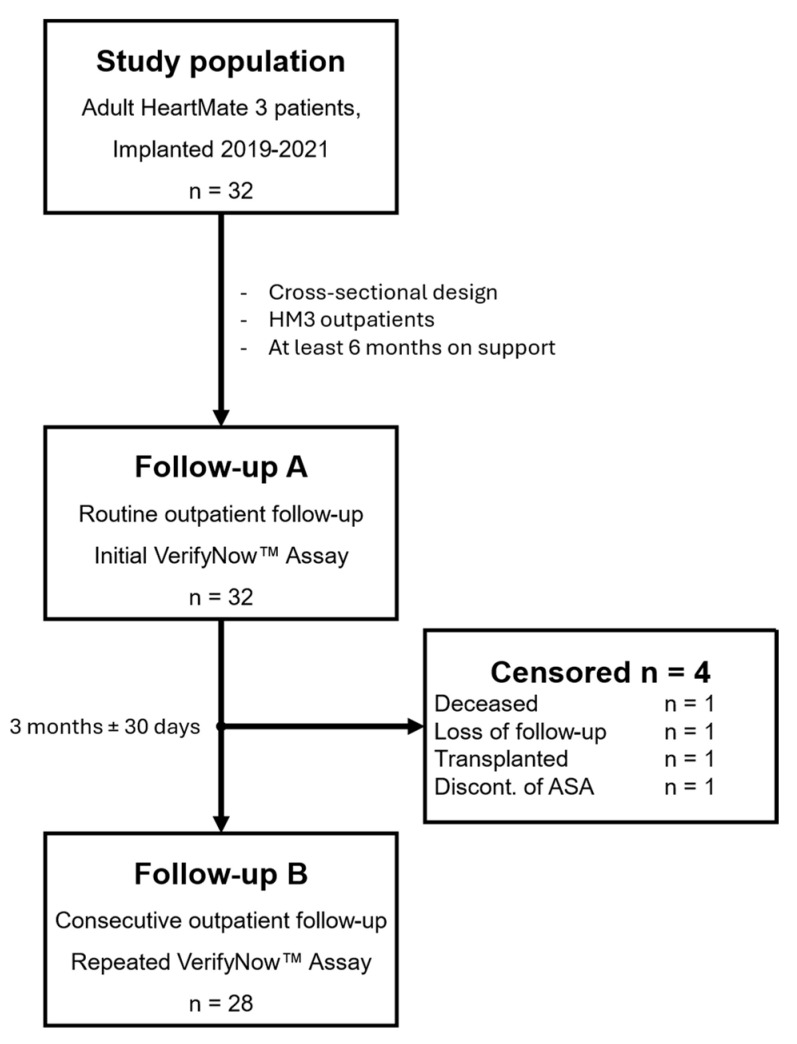
Study population; HM3 = HeartMate 3, ASA = acetylsalicylic acid.

Patients underwent two ASA platelet sensitivity assays (VerifyNow assay) during two consecutive outpatient follow-up visits (Figure 1). Platelet inhibition was measured using VerifyNow Aspirin Reaction Units (ARU). Patients with an ARU below 550 were classified as ASA responders, whereas those with an ARU of 550 or higher were classified as ASA-resistant [21,22].

### 2.2. Study Design

The primary endpoint was to investigate the incidence of HRAEs in ASA responders and non-responders assessed at two consecutive follow-up visits (Follow-ups A and B). The observation period of the study included the six months (180 days) prior to the first ARU assessment (Follow-up A) as well as the time between Follow-up A and the subsequent assessment (Follow-up B). During follow-up, all neurological events (including ischemic and hemorrhagic strokes), non-surgical bleeding (e.g., gastrointestinal bleeding), and suspected or confirmed pump thrombosis, were defined as any HRAE [3]. Secondary endpoints included temporal changes in ASA sensitivity, and potential correlations with baseline demographics, comorbidities, and concurrent medications.

### 2.3. Statistical Analysis

Statistical analysis was conducted using SPSS for Windows, version 29.0.0.0 (IBM, New York, NY, USA). Descriptive statistics are presented as mean ± standard deviation or median (IQR) for continuous variables, depending on their normal distribution as assessed by the Shapiro–Wilk test, and as a number (percentage) for categorical variables. For the comparison of continuous variables between ASA responders and ASA non-responders, the unpaired t-test or the Mann–Whitney U test was applied depending on the normality of the distribution, while the Fisher exact test was used to assess statistical significance for categorical data. Associations between two continuous variables were evaluated using Pearson’s correlation for normally distributed data and Spearman’s correlation for data that did not follow a normal distribution. For the comparison of variables assessed at Follow-ups A and B, either the paired *t*-test or the Wilcoxon test was used for continuous data, while McNemar’s test was applied for categorical data. Time-to-event analysis (cumulative incidence for any HRAE) was performed using Kaplan–Meier curves to compare ASA responders and ASA non-responders, with comparisons based on the log-rank test. Patients were censored in cases of heart transplantation, death, or loss of follow-up. Statistical significance was defined as *p* < 0.05.

## 3. Results

### 3.1. Baseline Demographics

Preoperative patient characteristics and comorbidities for the entire study population (n = 32), as well as stratified by ASA non-responders (n = 13) and responders (n = 19) during Follow-up A, are summarized in Table 1. The mean age of the patients was 59.0 ± 10.0 years, with a median body mass index (BMI) of 29.7 (26.8; 31.4) kg/m^2^; 15.6% of the patients were female. Ischemic cardiomyopathy was present in 51.7% of the patients, while 34.5% had dilated cardiomyopathy. The LVAD implantation strategy was a bridge to candidacy in 66.6% of cases and destination therapy and a bridge to transplant in 16.7%, respectively.

### 3.2. Primary and Secondary Endpoints 

There were no significant differences in baseline demographics and risk factors (Table 1) or in medications and pump parameters (Table 2). ASA non-responders and responders received comparable daily doses of ASA per protocol (*p* = 0.82, Table 2). At the first follow-up, conducted at POD 281 (193; 533), the median ARU was 535 (452; 568) (Table 3). Among the patients, 13 (40.6%) were classified as ASA-resistant, while 19 (59.4%) were identified as ASA responders (Table 1). The ARU measured at Follow-up A was statistically significantly higher in ASA non-responders (571 ± 13) compared to responders (468 ± 50, *p* < 0.001, Table 2).

**Figure 2:**
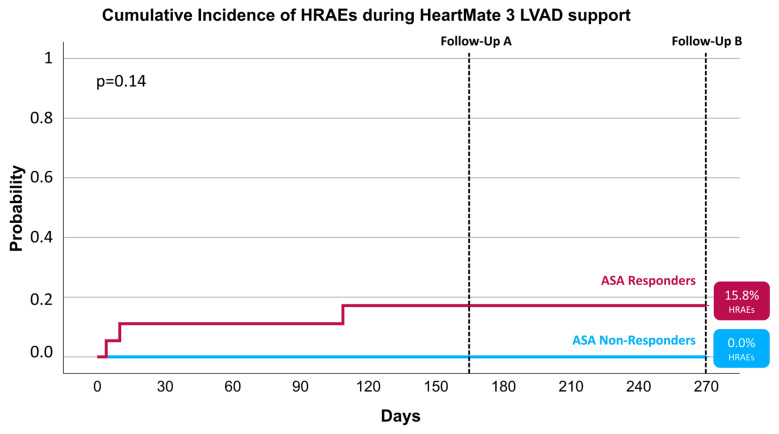
Cumulative incidence of hemocompatibility-related adverse events (HRAEs) during HeartMate 3 left ventricular assist device (LVAD) support stratified by acetylsalicylic acid (ASA) response type.

The cumulative incidence of HRAEs (Figure 2, Table 4) was numerically lower, but not statistically significantly different between ASA non-responders and responders 6 months before the first measurement (0.0% vs. 15.8%, *p* = 0.14), and no further HRAEs occurred between the two follow-up examinations.

A total of 28 patients underwent a second follow-up on POD 497 (437; 558), which occurred 106 (91; 133) days after the first follow-up. At Follow-up B, 20 patients (71.4%) were identified as ASA responders, while 8 (28.6%) were classified as non-responders (Table 2). 

**Figure 3:**
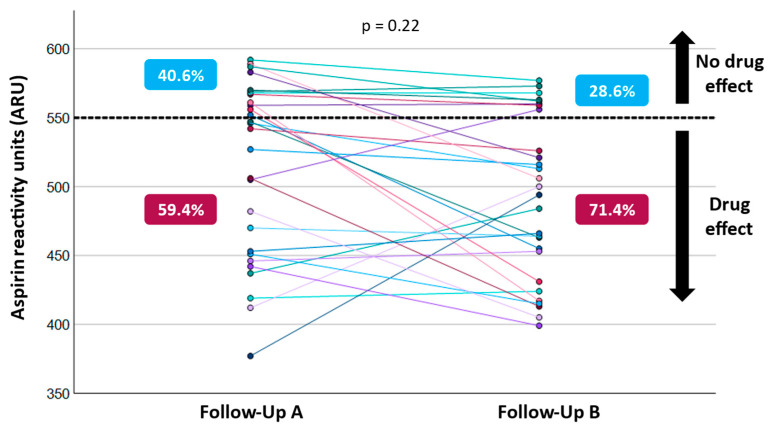
Temporal changes in measured Aspirin Reactivity Units (ARU) at Follow-Up A and B.

The overall distribution of ASA non-responders vs. responders did not change significantly over time (*p* = 0.22, Figure 3); however, ARU values measured at Follow-up B were significantly lower compared to earlier measurements (*p* = 0.03, Table 3).

Of the 13 ASA non-responders (40.6%) at Follow-up A, 7 (53.8%) remained resistant at the second ARU assessment. Additionally, 15 (78.9%) of the initial 19 ASA responders continued to exhibit aspirin sensitivity. Overall, 22 patients (68.8%) maintained a consistent ASA response between the two consecutive follow-ups. However, five patients (15.6%) transitioned from ASA resistance to sensitivity, while one patient (3.1%) shifted from being an ASA responder to a non-responder.

No significant correlations were observed between the ARU measured at the first follow-up visit and the ASA dose per day (r = 0.1, *p* = 0.57), BMI (r = −0.03, *p* = 0.87), INR (r = 0.05, *p* = 0.79), or lactate dehydrogenase (LDH) levels (r = 0.01, *p* = 0.97).

## 4. Discussion

Despite the success of the HM3 LVAD in nearly eliminating pump thrombosis and achieving overall superior hemocompatibility outcomes, as shown in the five-year analysis of the MOMENTUM 3 trial [4], challenges remain. The 2023 Society of Thoracic Surgeons INTERMACS annual report demonstrated that, over a five-year period, freedom from gastrointestinal bleeding (72% vs. 60%, *p* < 0.0001) and stroke (87% vs. 67%, *p* < 0.0001) was significantly higher in patients supported by the HM3 compared to those with non-fully magnetically levitated LVADs [1]. These persistent complications point to ongoing issues with bleeding and thrombotic events in LVAD patients, despite advancements in device technology.

This raises important questions about the role of pharmacological strategies, particularly antiplatelet therapy, in managing HRAEs in LVAD patients. Chronic aspirin therapy is widely used in this population to reduce the risk of thromboembolic events, particularly in patients still supported by HVAD or HMII devices. The most recent ISHLT guidelines [10] continue to recommend aspirin at a daily dose of 81–325 mg, in combination with VKAs, with a target INR range of 2.0–3.0. However, the benefits of aspirin therapy in HM3 patients have been increasingly questioned. For instance, the ARIES trial, led by Mehra et al. [23], demonstrated better outcomes in HM3 patients managed with a placebo compared to those receiving aspirin, particularly with regard to reduced bleeding without an associated increase in thromboembolism risk. This finding challenges the conventional assumption that aspirin is beneficial in this patient population and suggests that the role of aspirin therapy in HM3 patient management should be reconsidered.

In light of these considerations, this study aimed to assess the relationship between ASA responsiveness, as determined by VerifyNow assays [19], and the occurrence of HRAEs in patients with HM3 LVADs. Our findings showed a numerically lower, though not statistically significant, incidence of HRAEs (Figure 2) between ASA non-responders and responders (0.0% vs. 15.8%, *p* = 0.14). These results further suggest that aspirin responsiveness may not be a critical factor in hemocompatibility outcomes for HM3 patients.

This aligns with previous studies [11,23,24] exploring the impact of antiplatelet therapy in HM3 patients, where the benefits of ASA therapy have remained unclear. Additionally, a recent meta-analysis [25] compared VKA monotherapy to the standard combination [10] of a VKA and ASA. The analysis showed a significant reduction in HRAEs, particularly bleeding events with VKA monotherapy, without a concomitant increase in thrombotic events [25].

Other measurement techniques, beyond VerifyNow, have yielded similar findings. For example, a retrospective study by Rao et al. [26] evaluated patients who received less than a low dose of aspirin (<81 mg) and assessed the occurrence of thromboembolic events, alongside measuring ASA sensitivity via TEG. The study concluded that no thromboembolic events occurred, regardless of ASA sensitivity, suggesting that even very low-dose ASA therapy might be safe [26]. Furthermore, variations in the effects of ASA on platelet activation have been observed. Gum et al. [27] analyzed 325 stable cardiovascular patients treated with 325 mg ASA per day for more than seven days, comparing two methods of assessing aspirin resistance: optical platelet aggregation vs. PFA-100. They found that 5.5% of the patients were classified as ASA non-responders by the optical aggregation method, while 9.5% were non-responders according to PFA-100 measurements [27].

The use of various methods and principles to assess platelet inhibition presents a challenge in standardizing both results and their interpretation. Although VerifyNow and LTA rely on similar mechanisms, the correlation and agreement in identifying ASA-resistant patients have shown variability across studies. For instance, Coleman et al. [28] reported a strong correlation between VerifyNow and LTA, using EPI as the platelet-activating agent (r = 0.90). In contrast, a study by Lordkipanidze et al. [29] found only a weak correlation between the two methods (r = 0.13, *p* = 0.06). One potential reason for these discrepancies is the threshold of 550 ARU for defining ASA resistance in the VerifyNow assay. Paniccia et al. [16] observed that lowering this cutoff to 495 ARU improved agreement (κ = 0.42 vs. κ = 0.44), suggesting that threshold adjustments could influence assay concordance.

In the broader context of platelet sensitivity testing, similar results were observed regardless of the method employed. Flieder et al. [24] evaluated ASA resistance in 132 LVAD patients using LTA and/or impedance aggregometry (IPA), which measures platelet aggregation by adding an agonist to diluted whole blood and monitoring changes in electrical resistance between electrodes. Regardless of the method used, they found no significant difference in bleeding occurrence between ASA non-responders and responders, consistent with our findings using the VerifyNow assay. Regardless of the method used, multiple studies have reported ASA resistance, with findings indicating that 5–45% of patients may not achieve the expected platelet-inhibiting effect [13,14,15,26,27]. Our study similarly observed ASA non-responder rates, with no statistically significant temporal differences (Follow-up A: 40.6% vs. Follow-up B: 28.6%, *p* = 0.22). 

The mechanisms underlying ASA resistance remain incompletely understood [30]. However, several common contributing factors have been identified [31], including patient non-compliance [32], genetic polymorphisms (e.g., COX-1 variants) [33], hyper-reactivity of platelets [34], drug interactions [31], as well as conditions such as obesity [35] and diabetes [36], which may alter aspirin’s efficacy. 

Genetic polymorphisms, including variations in the PTGS1/COX-1 and PTGS2/COX-2 genes, have been linked to reduced antiplatelet efficacy, thereby increasing the likelihood of developing ASA resistance [37].

Despite differences in their mechanisms of action, other concurrent antiplatelet drugs can significantly impact platelet reactivity testing, as demonstrated by Kupó et al. in patients receiving tirofiban as part of concurrent antiplatelet therapy [38]. However, these interactions are not applicable to our findings, as none of the patients in our study were on dual antiplatelet therapy. 

Other concurrent medications, such as ibuprofen or indomethacin, both non-steroidal anti-inflammatory drugs, can contribute to ASA resistance by restricting the access for ASA to the COX-1 binding site [31].

Certain comorbidities, such as diabetes and obesity, can promote a prothrombotic state characterized by increased platelet reactivity, which in turn, reduces platelet sensitivity to ASA and other antiplatelet drugs [35,36]. Platelet and leucocyte counts, as well as hemoglobin and hematocrit levels, have been identified as factors influencing platelet reactivity testing [39]. In patients undergoing percutaneous coronary intervention who were treated with dual antithrombotic therapy, lower P2Y12 reaction units were correlated with higher hemoglobin and hematocrit levels [39]. Furthermore, comparisons of monocyte-platelet aggregation, platelet reactivity assessed by VerifyNow, and LTA between anemic and non-anemic patients revealed that lower hemoglobin levels were associated with increased platelet aggregation. This further supports hemoglobin as a predictive factor for platelet sensitivity [40].

### Limitations

Several limitations of this study should be acknowledged. First, this study was conducted at a single center with a relatively short follow-up period, which may limit the generalizability of the findings to other clinical settings. Second, as a cohort study, this research is observational in nature and cannot establish causation, making it susceptible to potential confounding factors. Specifically, variations in the duration of HM3 support at the time of ASA response assessment may have influenced results, as patients were at different stages of postoperative recovery and medication stabilization. Additionally, since patients were assessed as outpatients, potential interactions with other medications could not be fully controlled or excluded, possibly affecting the observed ASA response. For instance, the potential interactions between concurrent anticoagulation therapy and ASA, as well as their impact on INR levels, were not specifically evaluated. However, given that no significant differences in concurrent HF medication were observed between ASA non-responders and responders (Table 2), and considering that our center adheres to a standardized antithrombotic therapy regimen involving ASA and phenprocoumon, it is reasonable to conclude that these interactions are unlikely to have significantly influenced our findings. Third, the study’s relatively small sample size limits the statistical power and the robustness of the findings. Fourth, variations occurred in the timing of the ARU assessment following blood collection, as these assessments were conducted as part of routine clinical testing. However, the study complied with the manufacturer’s guidelines [22], which recommend initiating the VerifyNow assay between 30 and 240 min post-collection. Fifth, although correlations have been observed among the results of different platelet sensitivity assays, variability in findings may occur depending on the method employed. Since this study evaluated ASA resistance using the VerifyNow assay, the results may differ if alternative platelet sensitivity assays were utilized. Finally, as all patients in this study were HM3 recipients, the findings may not be applied to other devices or future LVAD generations; however, although not directly analogous to the ARIES trial [23], these results contribute to the growing body of evidence questioning the utility of aspirin in HM3 patients and should be interpreted with these limitations in mind.

## 5. Conclusions

Our findings provide evidence that ASA responsiveness, as assessed by the VerifyNow assay, does not significantly affect the risk of HRAEs in HM3 patients. These findings, along with those from the ARIES study, indicate a paradigm shift, warranting a thorough reevaluation of ASA use in this patient population.

## Figures and Tables

**Table 1:** Baseline characteristics of the study population, stratified by ASA response type during the first follow-up visit; ASA = acetylsalicylic acid, BMI = body mass index, Temp. RVAD = temporary right ventricular assist device.

Variable n (%), Mean ± SD or Median (IQR)	Entire Cohort (n = 32)	ASA Non-Responders (n = 13)	ASA Responders (n = 19)	*p*-Value
**Patient characteristics**				
Gender male female	27 (84.4%) 5 (15.6%)	10 (76.9%) 3 (23.1%)	17 (89.5%) 2 (10.5%)	0.37
Age (years)	59.0 ± 10.0	56.4 ± 11.9	60.6 ± 8.2	0.24
BMI (kg/m^2^)	29.7 (26.8; 31.4)	30.4 (28.0; 32.3)	29.3 (23.8; 31.1)	0.36
Blood Type 0 A B AB	14 (43.8%) 11 (34.4%) 4 (12.5%) 3 (9.4%)	8 (61.5%) 3 (23.1%) 1 (7.7%) 1 (7.7%)	6 (31.6%) 8 (42.1%) 3 (15.8%) 2 (10.5%)	0.78
INTERMACS Level 1 2 3 4 5	10 (34.5%) 5 (17.2%) 7 (24.1%) 6 (20.7%) 1 (3.4%)	6 (50.0%) 2 (16.7%) 2 (16.7%) 2 (16.7%) 0 (0%)	4 (23.5%) 3 (17.6%) 5 (29.4%) 4 (23.5%) 1 (5.9%)	0.70
Underlying disease Ischemic dilated unknown ischemic and dilated	15 (51.7%) 10 (34.5%) 2 (6.9%) 2 (6.9%)	5 (45.5%) 4 (36.4%) 2 (18.2%) 0 (0%)	10 (55.6%) 6 (33.3%) 0 (0%) 2 (11.1%)	0.35
Indication Bridge to candidacy Bridge to transplantation Destination therapy	20 (66.6%) 5 (16.7%) 5 (16.7%)	8 (66.7%) 2 (16.7%) 2 (16.7%)	12 (66.7%) 3 (16.7%) 3 (16.7%)	1.0
Surgical Access Sternotomy Thoracotomy + Hemisternotomy	25 (78.1%) 7 (21.8%)	13 (100%) 0 (0%)	12 (63.2%) 7 (36.8%)	0.07
Temp. RVAD, yes	12 (37.5%)	7 (53.8%)	5 (26.3%)	0.15
Concurrent valve procedures, yes	11 (34.4%)	7 (53.8%)	4 (21.1%)	0.07
Type of concurrent valve procedure: Mitral valve reconstruction Aortic valve replacement Tricuspid valve reconstruction Mitral + Tricuspid valve reconstr.	4 (12.5%) 2 (6.3%) 2 (6.3%) 3 (9.4%)	4 (30.8%) 1 (7.7%) 1 (7.7%) 1 (7.7%)	0 (0%) 1 (5.3%) 1 (5.3%) 2 (10.5%)	0.36
**Preoperative Comorbidities**				
Coronary Heart Disease	16 (50.0%)	7 (53.8%)	9 (47.4%)	1.0
Heart Attack	12 (37.5%)	4 (30.8%)	8 (42.1%)	0.71
Arterial Hypertension	15 (46.9%)	5 (38.5%)	10 (52.6%)	0.49
Pulmonary Hypertension	1 (3.1%)	0 (0%)	1 (5.3%)	1.0
Diabetes mellitus	8 (25.0%)	4 (30.8%)	4 (21.1%)	0.68
Atrial fibrillation	8 (25.0%)	3 (23.1%)	5 (26.3%)	1.0
Stroke History	4 (12.5%)	1 (7.7%)	3 (15.8%)	0.63

**Table 2:** Comparison of the variables assessed during the two follow-up visits of ASA responders and non-responders; ASA = acetylsalicylic acid, ACE-Inhibitor = Angiotensin-Converting Enzyme inhibitor, AT1-Antagonist = Angiotensin II Receptor Type 1 antagonist, BMI = body mass index, MAP = Mean arterial pressure, PI = Pulsatility Index, INR = International normalized ratio, LDH = lactate dehydrogenase, HI = hemolysis index.

	Follow-Up A			Follow-Up B		
Variable n (%), Mean ± SD or Median (IQR)	ASA Non-Responders (n = 13) 40.6%	ASA Responders (n = 19) 59.4%	*p*-Value	ASA Non-Responders (n = 8) 28.6%	ASA Responders (n = 20) 71.4%	*p*-Value
Aspirin Reactivity Units	571 ± 13	469 ± 50	<0.001	565 ± 7	463 ± 43	<0.001
Medications						
ASA/day (mg)	100 ± 0	105 ± 23	0.82	100 ± 0	105 ± 22	0.55
Inotropes, yes	0 (0%)	0 (0%)	-	0 (0%)	0 (0%)	-
Beta-Blockers, yes	12 (92.3%)	18 (94.7%)	1.0	8 (100%)	19 (95%)	1.0
ACE-Inhibitors, yes	5 (38.5%)	7 (36.8%)	1.0	2 (25%)	10 (50%)	0.40
AT1-Blockers, yes	7 (53.8%)	8 (42.1%)	0.72	5 (62.5%)	8 (40%)	0.41
Diuretics, yes	8 (61.5%)	13 (68.4%)	0.14	5 (62.5%)	11 (55.0%)	0.63
Proton pump inhibitors, yes	13 (100%)	19 (100%)	-	8 (100%)	20 (100%)	-
Demographic data						
BMI (kg/m^2^)	30 (28; 32)	29 (24; 31)	0.36	28.7 ± 4.8	30.6 ± 5.6	0.41
Weight (kg)	94 ± 11	93 ± 24	0.86	93 ± 11	98 ± 21	0.54
Hemodynamic variables and pump parameters						
MAP (mmHg)	79 ± 10.0	74 ± 13	0.29	72 ± 10	78 ± 12	0.22
Pump parameters Speed (rpm) Power (W) Flow (lpm) PI	5400 (5325; 5600) 4.1 (3.9; 4.5) 4.8 (4.4; 4.9) 3.4 (2.9; 4.4)	5400 (5200; 5600) 4.1 (3.8; 4.4) 4.7 (4.1; 5.1) 3.6 (3.0; 5.4)	0.95 0.80 0.67 0.65	5350 ± 200 4.0 ± 0.4 4.7 ± 0.7 2.8 (2.3; 4.4)	5390 ± 286 4.1 ± 0.4 4.5 ± 0.7 3.7 (2.9; 5.3)	0.72 0.49 0.50 0.10
Laboratory parameters						
INR	2.5 ± 0.3	2.5 ± 0.3	0.85	2.3 (2.1; 2.6)	2.3 (2.0; 2.6)	0.79
LDH (U/l)	230 ± 44	229 ± 75	0.42	198 (191; 220)	193 (181; 227)	0.47
HI	4.2 ± 2.9	9.6 ± 12.1	0.31	6.8 ± 6.7	5.0 ± 3.4	0.37

**Table 3:** Comparison of the variables assessed during Follow-up A and B; ARU = Aspirin Reactivity Units, ASA = acetylsalicylic acid, BMI = body mass index, MAP = Mean arterial pressure, INR = International normalized ratio, LDH = lactate dehydrogenase, HI = hemolysis index.

Variable n (%), Mean ± SD	Follow-Up A (n = 32)	Follow-Up B (n = 28)	*p*-Value
ASA responders	19 (59.4%)	20 (71.4%)	0.22
ARU	535 (452; 568)	497 (437; 558)	0.03
Postoperative Day	281 (193; 533)	421 (331; 663)	<0.001
ASA/day (mg)	100 ± 0	100 ± 0	1.0
BMI (kg/m^2^)	29.7 (26.8; 31.4)	30.6 (27.1; 33.0)	0.09
Weight (kg)	92 (78; 103)	97 (83; 106)	0.05
MAP (mmHg)	76 ± 12	77 ± 12	0.65
INR	2.5 (2.4; 2.8)	2.3 (2.1; 2.6)	0.06
LDH (U/L)	216 (181; 259)	196 (185; 220)	0.12
HI	4.0 (2.0; 8.8)	4.0 (2.0; 8.0)	0.78
Pump parameters Speed (rpm) Power (W) Flow (lpm) Pulsatility Index	5400 (5225; 5600) 4.1 (3.8; 4.4) 4.6 ± 0.6 3.4 (3.0; 4.8)	5400 (5225; 5600) 4.1 (3.9; 4.3) 4.6 ± 0.7 3.3 (2.7; 5.0)	0.01 0.19 0.88 0.30

**Table 4:** Comparison of the incidence of HRAEs between ASA responders and non-responders; HRAE = hemocompatibility-related adverse event, ASA = acetylsalicylic acid.

HRAEs	Total (n = 32)	ASA Non-Responders (n = 13)	ASA Responders (n = 19)	*p*-Value
Any HRAEs, yes	3 (9.4%)	0 (0%)	3 (15.8%)	0.25
Any Stroke, yes	1 (3.1%)	0 (0%)	1 (5.3%)	1.0
Ischemic Stroke, yes	1 (3.1%)	0 (0%)	1 (5.3%)	1.0
Hemorrhagic Stroke, yes	0 (0%)	0 (0%)	0 (0%)	-
Bleeding, yes	2 (6.3%)	0 (0%)	2 (10.5%)	0.50
Pump Thrombosis, yes	0 (0%)	0 (0%)	0 (0%)	-

## Data Availability

Data are contained within the article.
